# Association between Dietary Calcium Intake and Adiposity in Male Adolescents

**DOI:** 10.3390/nu11071454

**Published:** 2019-06-27

**Authors:** Jaak Jürimäe, Evelin Mäestu, Eva Mengel, Liina Remmel, Priit Purge, Vallo Tillmann

**Affiliations:** 1 Institute of Sport Sciences and Physiotherapy, University of Tartu, 51007 Tartu, Estonia; 2 Children‘s Clinic of Tartu University Hospital, 50406 Tartu, Estonia; 3 Institute of Clinical Medicine, University of Tartu, 50406 Tartu, Estonia

**Keywords:** dietary calcium intake, adolescent boys, adiposity, percentage of body fat, insulin resistance, adipocytokines

## Abstract

The aim was to investigate the possible association of dietary calcium intake with adiposity, insulin resistance, and adipocytokine values in adolescent boys. In this cross-sectional study, participants were 123 adolescent boys aged 13–15 years, who were divided into tertiles according to their dietary calcium intake. Dietary calcium intake was assessed using three 24 h dietary recalls. In addition, energy intake, body composition, physical activity (PA), and blood biochemical values were also measured. Mean body fat%, fat mass (FM), trunk FM, trunk fat%, and leptin differed between high and low tertiles of calcium intake after adjustment for age, pubertal stage, and PA. For the entire cohort, mean calcium intake was 786 ± 380 mg/day and was related to body mass index (BMI), FM, and trunk fat% but not to insulin resistance or adipocytokine values after adjusting for possible confounders. In addition, only 15.4% of the participants obtained or exceeded their mean dietary calcium intake requirements. These subjects who met their dietary calcium intake had significantly lower body fat% in comparison with subjects not meeting their dietary calcium intake. Odds ratio of being in the highest tertile of FM, trunk FM, and trunk fat% was 3.2–4.4 (95% confidence interval 1.19–12.47; *p* < 0.05) times higher for boys in low calcium intake tertile, compared to those boys in high calcium intake tertile. In conclusion, dietary calcium intake is inversely associated with total body and abdominal adiposity values in a specific group of healthy male adolescents with different body mass values.

## 1. Introduction

Obesity during childhood has become a major health issue that can lead to several chronic diseases and health problems later in life [1]. While some plateauing or decline in the prevalence of obesity has been observed in younger children [2], it is not the case in adolescents [1]. Childhood obesity appears to be the most common cause of insulin resistance in children and adolescents [3], and has been associated with type 2 diabetes mellitus, dyslipidemia, atherosclerosis, and coronary artery diseases [4] as well as with increased morbidity and mortality in later adulthood [5]. Adolescence is recognized as a critical period for the development of obesity [6]. Accordingly, it is important to identify modifiable risk factors during adolescence to prevent lifelong diseases related to obesity.

These modifiable risk factors include dietary habits, as dietary intervention is an important aspect in the treatment of obesity [7]. While it is commonly accepted that various combinations of macronutrients are able to regulate body mass, there is a need to further explore the effects of different micronutrients on body adiposity values [3,7,8]. Among different micronutrients, calcium and vitamin D intake may influence obesity and other health characteristics [9]. Studies with adults have reported inverse associations of calcium intake with body mass index (BMI) [10], body fat% [9], body fat mass (FM) [11], waist circumference [12], insulin resistance [13], and systemic inflammation [14] values. However, only few investigations have examined the association between dietary calcium intake and body adiposity values in children and adolescents with conflicting results [7]. There are studies to suggest that daily calcium intake may play a role in the modulation of body fat during growth and maturation [15,16], while other investigations have not found a relationship between daily calcium intake and body adiposity values in children and adolescents [17,18].

The association between dietary calcium intake with adiposity, insulin resistance, and adipocytokine values during growth and maturation has not yet been clarified. Accordingly, the aim of the present study was to investigate the possible association of daily calcium intake with adiposity, insulin resistance, and adipocytokine values in a specific group of healthy adolescent boys with a wide range of BMI values. It was hypothesized that adolescent boys with higher calcium intake would have lower body fat, insulin resistance, and adipocytokine values compared with adolescent boys with lower calcium intake values.

## 2. Material and Methods

### 2.1. Participants

The participants of the study were 123 pubertal boys aged between 13 and 15 years from different schools in Tartu, Estonia. The inclusion criteria for current study were that a boy had to be healthy and took part in obligatory physical education lessons at school. Participants were recruited as a part of larger longitudinal study. Boys using calcium supplementation were not included, nor were those taking any medications or having a clinical history of endocrine or metabolic problems, or cardiovascular, respiratory, or musculoskeletal diseases. All participants had their ordinary everyday diet. All procedures were approved by the Medical Ethics Committee of the University of Tartu, Estonia (Consent No 179/T-4, issue date 16.02.2009), and were explained to the boys and their parents who signed a consent form. Boys were divided in tertiles (low, medium, high) according to energy-adjusted calcium intake [16]. According to the previous study [19], where participants in the low calcium intake group (*n =* 26) compared with those in the high calcium intake group (*n =* 31) exhibited significantly higher values for BMI after adjustments for confounders, we should have had 50 participants per group to have a 0.80 chance (80% power) to detect the difference at 0.05 level of significance between the groups. In our study with 41 boys per group, we had 72% power to detect the difference of similar significance between the low calcium intake goup and high calcium intake group.

### 2.2. Maturity Assessment

Pubertal development was assessed by self-report using an illustrated questionnaire on pubertal stages according to Tanner [20]. Each boy was given line drawings, pictures, and descriptions representing genitalia and pubic hair development stages. The pubertal stage assessment according to the Tanner method, which uses the self-assessment of genitalia and pubic hair stage, has been previously validated [21] and used in our laboratory with previous studies with boys [22,23,24,25].

### 2.3. Anthropometry and Body Composition

Body mass and height were measured using calibrated medical digital scales (A&D Instruments, Abington, UK) and portable stadiometer (GMP anthropological instruments, Zurich, Switzerland) to the nearest 0.05 kg and 0.1 cm, respectively, with the participant wearing light clothing without shoes. Body mass index (BMI; kg/m^2^) was calculated as body mass in kg divided by squared height in meters. Whole body fat percentage, fat mass (FM), fat free mass (FFM), trunk FM, and trunk fat percentage were measured by dual-energy X-ray absorptiometry (DXA) using the DPX-IQ densitometer (Lunar Corporation, Madison, WI, USA) equipped with proprietary software, version 3.6. During DXA measurements, subjects were scanned in a light clothing while lying flat on their backs with arms on their sides. The medium scan mode and the standard subject positioning was used for whole body measurements, which were analyzed using the extended analysis option. The DXA measurements and results were evaluated by the same examiner. The precision of measurement expressed as coefficient of variation (CV) was less than 2% for all measurements [25].

### 2.4. Dietary Intake

Dietary intake was assessed using the average of three 24-h dietary recalls, including two weekdays (i.e., Thursday and Friday) and one weekend day (Saturday). Participants were asked to record everything they ate and drank for these days, and cup and bowl sizes were provided to help estimate portion sizes. The same dietitian interviewed all participants face-to-face and asked probing questions about their diet recalls. The nutrition data were entered into Nutridata System for Research (National Institute for Health Development, Tallinn, Estonia) and were analyzed for total daily energy (kcal/day), calcium (mg/day), and vitamin D (µg/day) intakes [22]. To estimate the prevalence of nutrient intake below or above the recommendations, the intakes of calcium and vitamin D were compared with their estimated average requirement (EAR), which are 1100 mg/day and 10 µg/day, respectively, according to the Institute of Medicine [26]. Finally, to control for confounding and mitigate for extraneous variation, calcium intake was adjusted for energy intake and expressed as mg per 1000 kcal consumed (i.e., energy-adjusted calcium intake) as suggested previously [27].

### 2.5. Physical Activity

The Actigraph uniaxial physical activity monitor (model GT1M; ActiGraph, Pensacola, CA), USA) was used to measure physical activity (PA) level and pattern for seven consecutive days. Epoch length was set at 15 s, and data were expressed as counts per min [23,28]. All participants were asked to wear accelerometer on the right hip, allowing removal for sleeping, bathing, and swimming. Physical activity was included for further analyses, if the participant had accumulated a minimum of 10 h of activity data for at least two weekdays and one weekend day [23,28].

### 2.6. Blood Analysis

After an overnight fast, between 08:00 and 09:00, a 10 mL blood sample was collected from the antecubital vein with the participant in an upright position. Blood serum was separated and then frozen at −80 °C for further analysis. Leptin was determined by radioimmunoassay (RIA) (Mediagnost Reutlingen, Germany) with intra- and inter-assay CVs <5%, and the least detection limit was 0.01 ng/mL. Adiponectin was also determined with a commercially available RIA kit (Linco Research, St. Charles, MO; USA). The intra- and inter-assay CVs were <7%, and the least detection limit was 1 µg/mL. Insulin was analyzed using Immulite 2000 (DPC, Los Angeles, USA). The intra- and inter-assay CVs for insulin were 4.5% and 12.2% at an insulin concentration of 6.6 µIU/mL, respectively. Glucose was measured with a commercial kit (Boehringer, Mannheim, Germany). Insulin resistance index was calculated using homeostasis model assessment (HOMA-IR): Fasting insulin (µIU/mL) × fasting glucose (mmol/L)/22.5 [29].

### 2.7. Statistical Analysis

Data analysis was performed using SPSS 25.0 for Windows (Chicago, IL, USA). Descriptive data are presented as means and standard deviations (SD). Boys were divided in tertiles according to energy-adjusted calcium intake [16]. Differences between tertiles (Low, Medium, High) groups were assessed by ANOVA using Bonferroni method. In addition, differences between tertiles were assessed after adjustment for age, pubertal stage, and total PA using univariate analysis of covariance (ANCOVA). Spearman correlation and partial correlation (controlling for age, pubertal stage, and total PA) coefficients were conducted to describe relationships of energy-adjusted calcium intake with body composition, insulin resistance, and adipocytokine values. Differences between EAR of calcium and vitamin D intake were assessed by ANOVA. Regression analyses were used to examine the relationships between energy-adjusted calcium intake and body fatness components through two separate models, where body fatness components were inserted as dependent variables and energy-adjusted calcium intake as independent variable. Model 1 was unadjusted model, while Model 2 was adjusted for age, pubertal stage, and total PA to determine the independent association with energy-adjusted calcium intake. Logistic regression analysis was also used to describe the odds ratio (OR) (95% confidence interval (CI)) of being at the highest tertile of each body fatness components according to the energy-adjusted calcium intake tertiles. Significance was set at *p* < 0.05.

## 3. Results

The study population included 123 adolescent boys, including 4 boys at pubertal stage 2, 30 boys at pubertal stage 3, 55 boys at pubertal stage 4, and 34 boys at pubertal stage 5, with the mean age of 13.9 ± 0.6 years. The mean BMI was 21.6 ± 5.4 kg/m^2^, with a range from 13.8 to 45.5 kg/m^2^ including 71% normal weight and 29% overweight/obese boys. Participants presented mean energy intake of 1798 ± 535 kcal/day, and the range was 622 to 3300 kcal/day. The mean calcium intake for all adolescent boys (*n =* 123) was 786 ± 380 mg/day, with a large range from 123 to 2460 mg/day. Data indicated that only 15.4% (*n =* 19) of the participants obtained or exceeded their calcium intake EAR [26]. The mean vitamin D intake was 3.2 ± 4.3 µg/day and only 8.1% (*n =* 10) of the participants met the recommended vitamin D intake EAR [26]. Boys who met their calcium intake EAR (*n =* 19) had a mean vitamin D intake of 5.1 µg/day, energy intake was 2330 kcal/day, and body fat% was 18.1 ± 9.1%, while boys who did not meet their calcium intake EAR (*n =* 104) had a mean vitamin D intake of 2.8 µg/day, their energy intake was 1700 kcal/day, and body fat% was 24.0 ± 12.%. These values were significantly different (*p* < 0.05) from the corresponding values in those boys who met their calcium intake EAR.

Body composition, PA, dietary intake, and blood biochemical characteristics of the study population are presented in Table 1. When the study participants were grouped by energy-adjusted calcium intake tertiles, body mass, and body adiposity values were lower with increasing tertile of calcium intake. The participants in the high calcium intake tertile had significantly lower body mass, BMI, body fat%, FM, FFM, trunk FM, and trunk fat% than those in the low calcium intake group. In addition, body fat%, FM, FFM, trunk FM, and trunk fat% remained significantly different between the high and low calcium intake tertiles after adjustment for age, pubertal stage, and total PA. No significant differences between different calcium tertiles were seen in blood biochemical characteristics. However, after controlling for confounding factors, participants in the high calcium intake tertile had significantly lower leptin levels when compared with the boys at the low calcium intake tertile. In addition, no significant differences between the three studied groups were seen in energy intake and vitamin D intake values (Table 1).

Bivariate correlation analysis revealed that calcium intake was inversely related to BMI, FM, trunk FM, and trunk fat% values (Table 2). The correlations between calcium intake and BMI, FM, and trunk fat% remained significant after adjusting for age, pubertal stage, and total PA values. In addition, calcium intake was correlated with body mass and FFM values after controlling for these confounding factors. In contrast, calcium intake was not associated with leptin, adiponectin, or insulin resistance (glucose, insulin, HOMA-IR) values (Table 2). Linear regression analysis showed that in unadjusted model (Model 1), the energy-adjusted calcium intake was negatively associated with trunk FM (β = −0.183; *p =* 0.043) (Table 3). After adjustment for age, pubertal stage and total PA (Model 2), the association slightly increased (β = −0.185; *p =* 0.042). There were no associations between calcium intake and other body fatness parameters in Model 1 as well as in Model 2 (*p* > 0.05) (Table 3). In addition, the OR of being in the highest tertile of FM was 4.4 (95% CI 1.55–12.47; *p =* 0.005) times higher for participants with low calcium intake tertile compared to those with high calcium intake tertile (Table 4). Similarly, the OR of being highest tertile of trunk FM and trunk fat% was 3.2 (95% CI 1.19–8.35; *p =* 0.021) and 3.3 (95% CI 1.19–9.05; *p =* 0.021) times higher, respectively, in the participants with low calcium intake tertile, compared to those with high calcium intake tertile (Table 4).

## 4. Discussion

This study was aimed to investigate the possible associations between calcium intake with adiposity, insulin resistance, and adipocytokine values in a heterogeneous group of 123 male adolescents with different adiposity values. An independent inverse association of calcium intake with different adiposity, but not with insulin resistance and adipocytokine values was observed in adolescent boys. Individuals with higher calcium intake had lower body adiposity values. Furthermore, those boys who met their calcium intake EAR had lower body fat% in comparison with those who did not met their daily calcium intake EAR. Accordingly, it could be suggested that adequate calcium intake is needed to maintain lower body fat values in adolescent boys during pubertal growth and maturation.

Our results demonstrated that lower calcium intake was associated with higher total body adiposity values in adolescent boys in this important developmental age, which is a critical period for the development of obesity [6]. These results are in accordance with a recent study in a large European cohort of children and adolescents [16]. In that study, an inverse correlation of total calcium intake with different adiposity indices was consistently observed in boys but not in girls, and the prevalence of obesity decreased significantly across tertiles of calcium intake [16]. While relatively few investigations have studied the role of calcium intake in modulating adiposity in children and adolescents [16], most of them have supported a significant association between calcium intake and adiposity measures [3,15,16,30], similarly to our results. In contrast, other studies have not found this relationship in children and adolescents [17,18,31]. The relationship between calcium intake and body adiposity has also been evaluated longitudinally demonstrating that lower calcium intake at baseline was associated with an increase in adiposity indices over a six-year period in boys with a wide range of BMI values but not in girls [16]. Similarly, calcium intake was not related to two-year change in body fat in lean peripubertal girls [17]. In addition to possible gender differences, it has been suggested that a certain threshold for calcium intake and/or BMI may be needed to observe the relationship between calcium intake and total body adiposity values [13]. It has also been argued that the effects of calcium intake on body adiposity depend on higher percentage of body fat in children [7]. In accordance with this, the OR being in the highest tertile of FM in our study was more than four times higher for participants with low calcium intake compared to those with high calcium intake and more than three times higher being in the highest tertile of trunk FM or trunk fat%.

Inverse association of calcium intake with abdominal adiposity was also identified in the current investigation, which is in accordance with the results of other studies carried out with 8–9-year-old [32], 9–12-year-old [17], and 12–19-year-old [3] children with various BMI values as well as 7–18-year-old children and adolescents with obesity [33]. Interestingly, Barr [17] demonstrated that calcium intake below the median was independently associated with higher trunk fat% in 9–12-year-old girls. Similarly, calcium intake was negatively correlated with trunk fat% after controlling for potentially confounding variables in our study. It has been suggested that trunk fat may be preferentially affected by calcium intake [17,34], and abdominal adiposity is closely related to metabolic syndrome, type 2 diabetes mellitus, and cardiovascular diseases [3,4].

Another important finding of the present study was the inadequate calcium intake in majority of studied adolescents. It appeared that only 15.4% (*n =* 19) met their daily calcium intake EAR, similarly to the results of other studies in children and adolescents [3,32,35]. In fact, most studies have observed that more than 90% of studied children and adolescents do not meet the required daily calcium intake [3,32,35]. Therefore, calcium appears to be one of the micronutrients with the highest rate of inadequate consumption worldwide [36]. In our study, adolescent boys with adequate calcium intake had significantly lower body fat% in comparison with adolescents with inadequate daily calcium intake.The importance of adequate calcium intake in adolescent boys should be emphasized because about 45% of bone mineral accrual occurs during early adolescence [37]. This age and maturation period provides a limited window of opportunity for maximizing peak bone mass [38], which is a major determinant of fractures, a major public health problem in adults [6].

Different mechanisms have been proposed to explain the association between calcium intake and body adiposity. For example, it has been suggested that adequate calcium intake increases the oxidative capacity of adipose tissue [32]. Higher calcium intake is associated with reduced 1,25-vitamin D levels, which in turn induce intracellular calcium content to decrease in adipose tissue, stimulating lipolysis, and inhibiting lipogenesis in the adipocyte [39]. In contrast, low calcium intake is associated with high intracellular calcium concentrations, which may then increase the rate of lipogenesis and inhibit lipolysis, resulting in increased adiposity [39]. Furthermore, dietary calcium intake may influence fat metabolism by increased faecal fat excretion [40] and decreased hunger or increased satiety [41]. Anti-obesity mechanisms of vitamin D have also been suggested [16], with vitamin D interacting in a complex system of absorptive and endocrine functions [10]. However, it has to be considered that all these suggested mechanisms still lack confirmation in human longitudinal studies [16].

There was no association between daily calcium intake and measured adipocytokine values in adolescent boys with various adiposity values. Therefore, leptin is synthesized by adipocytes and is directly related to the amount of body fat [42]. While calcium intake was related to body FM in current study, it would have expected to also observe an association between calcium intake and leptin values, similarly to a study with premenopausal women [13]. The differences between the results of our study and that of da Silva Ferreira et al. [13] could be explained by different leptin levels in these studies, premenopausal women having considerably higher leptin and adiposity values compared to adolescent boys in the present study. However, leptin concentration was significantly lower in high calcium intake tertile compared with leptin value in low calcium intake tertile after controlling for energy intake, PA and maturation values in adolescent boys, which was also observed in premenopausal women [13]. Further studies are needed before any conclusion about possible association of daily calcium intake with leptin can be drawn.

The possible relationship of calcium intake and insulin resistance has not yet been fully clarified. Unlike previously reported investigations in adults [13,43,44] and adolescents [28], no association between calcium intake and HOMA-IR as an index of insulin resistance was seen in our specific group of adolescent boys with different adiposity values. While dos Santos et al. [30] found a significant inverse association between calcium intake and HOMA-IR among obese but not normal weight adolescents. In accordance with our results, a recent study reported no relationship between calcium intake and HOMA-IR in adult males, while calcium intake was related to HOMA-IR in adult females [43]. Accordingly, the reported association between calcium intake and insulin resistance that has been found in adults [13,43,44], should be further investigated in adolescents using a direct measure of insulin resistance.

There are some limitations in our study that should be considered. Firstly, this is a cross-sectional study, which limits our ability to make causal inferences. In addition, although the same dietitian interviewed all participants and reviewed their diet recalls with each participant, self-reported dietary intakes are subject to error as it depends on the memory of adolescents, and is susceptible to under- and over-reporting. Another potential limitation is that some significant associations would have not been detected due to slightly underpowered study, although the number of participants was similar to previous cross-sectional studies in this area [3,9,13,19]. The specific information about socioeconomic status and/or maternal/paternal overweight/obesity was also not collected. Finally, our findings are limited to a specific group of Caucasian male adolescents with specific age and maturation range, and wide range of total body adiposity values.

This study has also some strengths. Firstly, objective assessment of PA by accelerometry and body composition by DXA were used in this study. In addition, the present study is one of the few studies that has evaluated possible associations of calcium intake with adiposity, insulin resistance, and adipocytokines taking into account several confounders including pubertal maturation, energy intake, and PA. These findings help to understand the possible associations between calcium intake levels and adiposity values during growth and maturation.

## 5. Conclusions

Dietary calcium intake is inversely associated with total body and abdominal adiposity values in a specific group of healthy male adolescents with different body mass values. In contrast, no relationships of calcium intake with adipocytokine and insulin resistance values were observed. In addition, adolescents with adequate calcium intake had significantly lower body fat% in comparison with those adolescents with inadequate daily calcium intake. Odds ratio of being in the highest tertile of FM, trunk FM, or trunk fat% was more than three times higher for participants in low calcium intake tertile compared to those with high calcium intake tertile.

## Figures and Tables

**Table 1:** Mean ( ± SD) descriptive characteristics of the studied sample according to tertiles of energy-adjusted calcium intake.

Variable	Total (*n =* 123)	Low Ca Intake Tertile (*n =* 41)	Medium Ca Intake Tertile (*n =* 41)	High Ca Intake Tertile (*n =* 41)
Age (yrs)	13.9 ± 0.6	13.9 ± 0.6	13.8 ± 0.6	14.1 ± 0.6 ^#^
Height (cm)	169.0 ± 8.6	170.7 ± 8.7	167.5 ± 8.4	168.9 ± 8.7
Body mass (kg)	62.5 ± 19.4	70.5 ± 24.1	58.7 ± 15.2 *	58.2 ± 15.4 *
BMI (kg/m^2^)	21.6 ± 5.4	23.8 ± 6.5	20.7 ± 4.3 *	20.3 ± 4.4 *
Overweight/obese (n/%)	36/29.3	16/39.0	12/29.3	8/19.5
Body fat%	23.1 ± 11.7	26.6 ± 12.1	22.3 ± 11.4 ^†^	20.3 ± 11.0 *^†^
FM (kg)	15.2 ± 12.3	19.8 ± 15.4	13.6 ± 9.4 *^†^	12.2 ± 10.0 *^†^
FFM (kg)	46.5 ± 10.0	49.8 ± 11.4	44.4 ± 8.6 *	45.3 ± 9.3 *^†^
Trunk FM (kg)	6.3 ± 5.6	8.5 ± 7.2	5.6 ± 4.3 *^†^	4.7 ± 4.4 *^†^
Trunk fat%	21.4 ± 12.1	25.3 ± 12.7	20.7 ± 11.7 ^†^	18.1 ± 11.0 *^†^
Total PA (counts/min)	398 ± 161	397 ± 165	403 ± 145	393 ± 174
Energy intake (kcal/day)	1798 ± 535	1785 ± 543	1769 ± 561	1839 ± 512
Calcium intake (mg/day)	786 ± 380	515 ± 189	738 ± 240 *	1104 ± 408 *^#^
Calcium intake (mg/1000 kcal)	434 ± 146	287 ± 60	418 ± 40 *	597 ± 98 *^#^
Vitamin D intake (µg/day)	3.2 ± 4.3	2.7 ± 3.0	3.5 ± 5.4	3.5 ± 4.1
Leptin (ng/mL)	6.1 ± 8.6	8.1 ± 11.2	5.6 ± 7.5	4.6 ± 6.2 ^†^
Adiponectin (µg/mL)	8.0 ± 4.4	7.5 ± 4.1	8.3 ± 4.3	8.3 ± 4.9
Glucose (mmol/L)	5.1 ± 0.4	5.1 ± 0.4	5.0 ± 0.4	5.1 ± 0.4
Insulin (µIU/mL)	14.2 ± 7.7	15.8 ± 8.5	12.7 ± 6.4	14.2 ± 7.9
HOMA-IR	3.2 ± 1.8	3.6 ± 1.9	2.9 ± 1.5	3.3 ± 1.9

* Significantly different from Low Ca intake tertile; *p* < 0.05; ^#^ Significantly different from Medium Ca intake tertile; *p* < 0.05; ^†^ Significantly different from Low Ca intake tertile after adjustment for age, pubertal stage, and total PA; *p* < 0.05.

**Table 2:** Correlation coefficients of energy-adjusted calcium intake with body composition and blood biochemical variables.

Variables	Bivariate Correlation	Partial Correlation Adjusted for Age, Pubertal Stage, and Total Physical Activity
Body mass (kg)	−0.157	−0.211 *
BMI (kg/m^2^)	−0.192 *	−0.205 *
Body fat%	−0.167	−0.132
FM (kg)	−0.195 *	−0.190 *
FFM (kg)	−0.104	−0.194 *
Trunk FM (kg)	−0.188 *	−0.150
Trunk fat%	−0.189 *	−0.199 *
Leptin (ng/mL)	−0.111	−0.103
Adiponectin (µg/mL)	0.072	0.130
Glucose (mmol/L)	0.022	0.033
Insulin (µIU	−0.061	−0.059
HOMA-IR	−0.064	−0.053

* Statistically significant; *p* < 0.05.

**Table 3:** Linear regression coefficients examining the associations of energy-adjusted calcium intake with body fatness components.

	Calcium Intake				
	B ± SE	95% CI	R^2^	β	*p* value
**Model 1**					
Body fat%	−0.01 ± 0.01	−0.03; 0.01	0.13	−0.128	0.157
FM	−14.60 ± 7.58	−29.61; 0.41	0.03	−0.172	0.056
Trunk FM	−7.10 ± 3.47	−13.97; −0.23	0.03	−0.183	0.043
Trunk fat%	−0.01 ± 0.01	−0.03; 0.003	0.02	−0.147	0.104
**Model 2**					
Body fat%	−0.01 ± 0.01	−0.03; 0.001	0.06	−0.125	0.168
FM	−14.70 ± 7.65	−29.84; 0.45	0.05	−0.174	0.057
Trunk FM	−7.20 ± 3.50	−14.13; −0.26	0.05	−0.185	0.042
Trunk fat%	−0.01 ± 0.01	−0.03; 0.003	0.05	−0.146	0.108

Model 1: Unadjusted model. Model 2: Adjusted for age, pubertal stage, and total PA. B—unstandardized coefficient. SE—standard error. CI—confidence interval. β—standardized coefficient.

**Table 4:** Odds ratios of highest tertile of body fat components versus other tertiles (medium+lower) of body fat components.

	Body Fat%			FM			Trunk FM			Trunk Fat%		
Ca tertiles	OR	95%CI	*p* value	OR	95%CI	*p* value	OR	95%CI	*p* value	OR	95%CI	*p* value
High	Ref			Ref			Ref			Ref		
Medium	0.92	0.36; 2.35	0.867	1.56	0.62; 3.93	0.342	1.97	0.78; 4.97	0.154	1.24	0.49; 3.12	1.24
Low	2.79	0.99; 7.83	0.051	4.39	1.55; 12.47	0.005	3.15	1.19; 8.35	0.021	3.29	1.19; 9.05	0.021

All models were adjusted for age, pubertal stage, and total PA.

## Abbreviations

BMI, body mass index; EAR, estimated average requirement; DXA, dual- energy X-ray absorptiometry; FM, fat mass; FFM, fat free mass; HOMA-IR, homeostasis model assessment of insulin resistance; OR, odds ratio; PA, physical activity.
